# A Medical Student’s Clinical Observations in the Two Schools of Medicine*Read before I. H. A., June, 1883.

**Published:** 1884-08

**Authors:** Rollin R. Gregg

**Affiliations:** Buffalo, N. Y.


### A MEDICAL STUDENT'S CLINICAL OBSERVATIONS IN THE TWO SCHOOLS OF MEDICINE.*

ROLLIN R. GREGG, M. D., BUFFALO, N. Y.

August, 1849, I began the study of medicine in Michigan with an allopathic physician of marked ability, large practice, and many years of experience. But notwithstanding his great experience, or, rather, no doubt, because of this, he frequently deprecated the uncertainties of medicine. Sometimes, indeed, his dissatisfaction was expressed in no very mild or measured terms.

Late in the fall of the same year, I received a letter from an uncle, the late Dr. Durfee Chase, of Palmyra, N. Y. (who had heard I had commenced the study of medicine), urging me to study Homœopathy, and telling me at the same time of the much greater certainties and satisfaction in its practice than in that of the old school. To give force to what he said, he referred to twenty years of extended experience in the practice of allopathy and ten years in the practice of Homœopathy, and said nothing could induce him to return to the old school methods of treating disease. Not long afterward I referred the matter to my old school preceptor to get his opinion, and, of course, received it in vigorous language, which was not complimentary to the new system of medicine.

Time wore on, and with it came frequent deprecations from

---

* Read before I. H. A., June, 1883.

my preceptor of unsatisfactory results in the treatment of even ordinary cases of disease, until, one day in the succeeding spring, he came into his office on his return from visiting a patient two or three miles distant, and seizing his saddle-bags in his right hand (from off the flexed left forearm, where he bore them very proudly when going out to or coming in from his carriage), threw them with all his force into the farthest corner of his office, and exclaimed: "There, —— you, lie there, I will never take you out of this office again as long as I live." His partner, who was present, and I both looked up in astonishment from our reading, and both asked: "Why, Doctor, what is the matter?" "Matter enough, —— it," was his vigorous response, and that was all we could get out of him for some time, until he became calmer. Then he said: "I was called yesterday afternoon to see Mr. ——, who had a simple attack of the bowels that any old woman could have cured in a few hours with a little herb tea, but I, like a fool, must give him some of my medicine, and now he is in a dying condition and will die to-night." Then he went on raving again in his chagrin and honest indignation at the result, and denouncing medicine as a system of barbarism, with no science, or truth, or certainty in it, as much more liable to kill than to cure, etc., etc.

After he was through I did not fail to remind him of the talks we had had of Homœopathy, and that I had better be looking into that, if such were the facts about his system of practice; but this only added fuel to the flame. Said he: "D—— Homœopathy, it is nothing but a system of quackery. All the traditions of medicine from Hippocrates down are with our school, and what knowledge is possessed in medicine, which is very little, is with us, and there is no other way but to hold on until something better comes," etc. True to his judgment, his patient did die that night; but not true to his own vigorous declarations, he was out himself, bearing his saddle-bags as proudly as ever, and visiting other patients before night of that same day. To do him justice, however, he appeared to shrink from going, for fear of the further mischief he might do, and expressed himself in some such manner.

In the meantime I had been having more correspondence with Dr. Chase about Homœopathy, asking questions as to its principles and what it could really do in serious forms of disease; if his experience in it had shown him that in similar cases it worked better results than allopathic medication, to all of which were received favorable and what appeared to be truthful replies.

During my study of allopathy, moreover, there was another thing that did not escape my attention. It was this: I met some of the ablest physicians in the State, and one of the foremost topics of conversation with them always was the uncertainties of medicine; with remarks to me personally that it was right and all very well for me to be enthusiastic then, and press on in my studies, but when I entered upon practice I would be brought face to face with the realities of medicine, which I would find far different from the theory, and the longer I practiced, the less would be my confidence in it. Repeatedly were these things said to me, and the older and the more experienced the physician, the more and stronger would he talk in this way.

The effect of all this talk upon a mental constitution averse to doing violence to the human system with crude or harsh medicines (some of which I had taken) finally was that in June, 1850, I packed my trunk and went after "that humbug, Homœopathy." And let me say here, in passing, that I was not long in satisfying myself with the change.

The first striking case I saw cured homœopathically, not long after entering Dr. Chase's office, was a very severe case of chorea in a girl of about twenty years. She had been suffering for a year or over, was pale and emanciated, and in almost constant motion; and the father told the doctor he had paid out over a hundred dollars for medical treatment for her, but she had continued getting steadily worse. The doctor prescribed Pulsatilla, 6th centesimal potency, made five prescriptions for her at intervals of one to two weeks, charged twenty-five cents for each prescription, and entirely cured the patient, at a total cost to the father of *one dollar and twenty-five cents.* My interest in the new system was then a little aroused, as may well be imagined.

The next striking effect I saw from homœopathic treatment was upon myself. For several years I had been attacked once or twice a year with a hard aching pain in the left leg from the thigh to the ankle, as though it were in the bones, always crippling me so that it was difficult to walk for a day or two. In the fall after entering Dr. Chase's office I was attacked with this pain; bore it part of one day and into the next, when I consulted the Doctor about it. He gave me a dose of Rhus tox., 6th centesimal potency, which relieved me entirely of pain in an hour or less; and in two or three hours large yellow pustules broke out upon the leg below the knee, and continued out two or three days, then dried up. From that day to this I have never had that pain or the least threatening of it.

There was not the slightest appearance of pustules on the leg at the time the dose of Rhus was taken, never had any appeared before—upon the relief of the pain or under any circumstances; and it was a great wonder to me then and for years thereafter how suppuration could have taken place in those pustules in two or three hours. It was entirely contrary to all the teachings of the books then and since about suppuration, and excited my interest. Twelve years afterward I found the solution of the mystery in the facts: that all pus corpuscles are nothing but decolorized blood corpuscles; that the cerum of the blood of all subjects having a tendency to tuberculosis is too watery; that when such is the case some of the blood corpuscles are decolorized prematurely in the circulation by the too watery serum and under the law of endosmosis; that my blood was at that time and had from childhood been too watery, hence some of these decolorized blood corpuscles were at all times circulating in my vessels; that the attacks of pain were due to a partial congestion of these decolorized corpuscles, or some of them, in the vessels of the bones of the leg to give me the pain; and that the dose of Rhus speedily relieved that congestion and forced those corpuscles to the surface to congest in the pustules; hence the suppuration in two or three hours. Those corpuscles were already decolorized and congested in the vessels of that leg, and all that was needed was to bring them to the surface, as they were by the action of Rhus, to give relief and to present the evidences of such hasty suppuration. Thus it is that simple and trifling facts in themselves overturn all the fine-spun and high-sounding theories that ignorance and error have been centuries in building. I have seen many evidences of similar rapid suppurations under the curative action of medicine in other cases since.

A few months after that I again had personal evidence of the speedy relief that the new system of medicine would afford in severe and serious suffering. After months of close confinement and very hard study, I was one day attacked with headache, not very severe at first, but which increased in severity as the day passed on, became worse in the evening, so that little sleep was obtained that night, and by the next morning reached such violence in darting pains through my temples when dressing that I could not remain up any longer. This was an entirely new experience for me, as I had not had pains of any kind in the head in years. The appetite was entirely gone, the tongue coated, and the only way I could endure the pain was by keeping very quiet. The Doctor, being informed I was ill, came in to see me, inquired into the particulars of the symptoms, gave me four or five small pellets of Bryonia, 6th centesimal potency, dry upon my tongue, stood by me five or ten minutes, when he stepped into another room—and where I followed him in less than another ten minutes almost wholly relieved from pain. And I never had that pain afterward, although I went on with my studies about as hard as ever that afternoon.

At the close of my first year's study of Homœopathy I returned home to Michigan on a visit to my parents. While there I heard that there was an epidemic of dysentery prevailing in an adjoining county, from which many were dying. This prompted me to leave some homœopathic remedies for dysentery in case any of the family should be attacked. I left Arsenicum and Nux vomica only in the 6th centesimal potency. Two weeks after leaving home to return to my studies, my father was taken down with what proved to be a terrible attack of that disease. He had no confidence in Homœopathy and sent for his family physician (who was my old allopathic preceptor) as soon as he was attacked. The disease increased in violence from day to day, until there was scarcely any sleep; evacuations of blood and mucus, with great suffering from tenesmus, every five to ten minutes night and day; high fever with tongue entirely dry and almost black, and other correspondingly severe and alarming symptoms. On the ninth or tenth day counsel was called, and little hope was given of his recovery. The next morning another consultation was held. The patient had passed a terrible night, and the physicians left the house at 11 A. M., after saying they could give no more encouragement in the case.

My mother had seen the danger for several days, and being a resolute woman when aroused, told my father that she was going to take the responsibility of stopping the allopathic treatment and depending upon the homœopathic remedies I had left. He pleaded with her not to do so, saying he was too ill and in too great danger to permit a change of medicine; but the change was made and one dose of four or five pills of Arsenicum given him upon his very dry tongue. In ten minutes he expressed himself as feeling much relieved, in fifteen minutes he fell asleep and slept as peacefully as a child for four hours. During his sleep he perspired so excessively as to saturate the bedding under him entirely through from his head to his feet—the first perspiration he had had from the beginning of his disease. On awakening he said he was entirely free from pain and greatly refreshed, partook of some broth with a relish, and that was the end of all dysenteric action in his case.

At 4 P. M. his physician called again to see him, and his first expression was: "For God's sake, what has been done here and what does this mean?" My mother said: "Why, Doctor, what is the matter?" "Why," he said, "I left this patient five hours ago a dying man, and now he is entirely out of danger and convalescence fully established; his fever is all gone; his tongue, from being so dry and black for days, has become moist and almost natural; and did I not know the fact, I could not have been made to believe he had been seriously sick." Then he further said "that in all his experience he had never seen anything like it, or that at all approached it," and again asked what it meant, or what could be the explanation of it. My mother then told him she stopped his medicine and gave one dose of homœopathic medicine I had left for dysentery soon after he left the house in the morning, and explained the quick effect it had in giving relief and putting the patient to sleep. Upon this the Doctor took his hat and left the house, and never called again. Convalescence was short, rapid, and complete, no further trouble of any kind arising in the case.

Perhaps it should be said, in partial explanation of the marvelous rapidity of relief and cure in this case, that my father was naturally a robust, strong man, with good powers of endurance. But there can be no doubt he would have died of his disease in two or three days at most but for that fortunate dose of Arsenicum and the stopping of violent medicine. He was then about sixty years of age and lived until his eighty-fourth year.

A month or six weeks after my return to my studies from that fortunate visit home, I was again astonished at the marvels Homœopathy will often accomplish when rightly handled. One very cool afternoon in July, so cool as to almost require a fire to keep us comfortable, Dr. Chase, another student, and myself, were sitting in the office reading, when two men entered to consult the Doctor. One of these was a dark-complexioned man, of black hair and eyes, and had a haggard and greatly distressed expression of countenance. He was a canal-boat captain and the other man was his steersman and came with him to take care of him and prevent his doing violence. The latter explained that the captain had been suffering from earache three or four days, had scarcely slept at all for two or three days and nights, and on his way down from Rochester that day had become delirious and driven his family out of the cabin, thrown furniture into the canal, and attempted other acts of violence. His pain was all confined to his left ear, if I remember rightly; he was writhing in agony, and there was not the slightest appearance yet of suppuration.

The Doctor gave him a few pellets of Chamomilla, 6th, dry upon his tongue. In five minutes he expressed a sense of great relief, in ten minutes he said he was wholly relieved and began to perspire; and notwithstanding the office was uncomfortably cool for the rest of us, I never before or since saw any man perspire so profusely as he did for an hour. The water ran down his face and neck in streams, his hair was saturated, and his clothes were drenched with it. Upon baring his breast and back the water was running in numerous streams half as wide as one's finger down his body. There had been no breaking of the abscess in the ear to afford this marvelous relief and account for the result, as we examined into that very carefully, and there was nothing else it could be attributed to but the effect of Chamomilla. Nor was he a strongly built or vigorous appearing man, to thus account for such a powerful reaction. On the contrary, he was a man of medium height, thin in flesh, and of a decidedly bilious appearance; so the medicine in this case must have the whole credit in the wonderful transformation. In less than two hours he left the office a fully restored man in mind and body, and as happy as only a person under such circumstances knows how to be. We heard from him afterward, and he had no further trouble.

Apropos to this case I will speak of another case of earache occurring in the first year of my practice. This was in a little girl aged four or five years, whom I attended forty-eight hours without relief, when she became frantic from pain, with screaming, rolling on the floor, and was becoming unmanageable. I had given her several remedies, including Chamomilla, with no effect, and at this juncture of such violence I gave her one dose of Coffea, 6th, which wholly relieved her in five to ten minutes, and she had no more pain afterward, notwithstanding the abscess in the ear went on gathering, but did not break until two days afterward, when it did and discharged quite profusely. Thus it is that Homœopathy frequently lifts us above suffering, while what the old school regards as the physical necessities for pain continue. Again, no longer ago than the spring of 1882, I relieved just as marvelously a delicate, sickly man, whom I had been treating two or three days for earache, left side, without relief, by giving him one dose of Arnica, 1000th potency, (Jenichen's), under the symptom of great soreness of the ear and whole side of the head affected. Nor in his case was there a breaking of an abscess to account for the effect. Entire relief came in half an hour or so, but no discharge.

Another proof of relief given by our remedies before the discharge of a gathering abscess it might be well to give in this connection. A month or two after I began the practice of medicine an editor consulted me for a felon upon one of his thumbs, from which he had been suffering greatly about a week. He told me he had scarcely slept at all for three or four days and nights. This was about seven P. M., and I gave him a dose of Phosphorus, 30th, and told him to go to bed as soon as he could reach his room. This he did, fell asleep at once, and did not wake until nine o'clock the next morning, when he found himself in the exact position he took on lying down the night before, apparently not having moved during the whole night. But his felon did not break to discharge the least until two days afterward.

But we must get back from this digression to student life, as that is our special theme now. And here is as good a place as any to tell of the remarkable difference I found in the talk of the few homœopathic physicians I met in those early times about the action of medicine, and the beautiful results they had seen in contrast with what I had heard so much of in the old school upon that subject. Those homœopathic physicians had all been allopathists before going into the new school, some of them having had much experience therein; and it was of no little interest to me to hear them talk of the old school exactly as I had heard it so often talked of before by those who were still in it and whose prejudices were all in its favor, and the enthusiasm of the former for the new school and what it could do. Again, when I met old school physicians, after beginning the study of Homœopathy, to talk with them of the action of medicine, it was the same old story with them, as it had been with others before, of the uncertainties of and dissatisfaction with the practice of medicine.

During the balance of my student life I saw much more, of course, of the marvelous reliefs given and great cures accomplished homœopathically than is above narrated; but as this paper is already getting lengthy, I must pass all that and hasten on to another personal experience that changed my whole current of thought in Homœopathy and left its impress with me for a lifetime.

From hard study and little out-door exercise I became greatly constipated. For this I consulted my preceptor, and he prescribed Nux vomica, 6th, three or four doses a day. There was, however, no satisfactory result from its action, and he then gave me Sulphur, 12th, alternately with Nux, two doses each day for a time. This had no better effect, and Sulphur was dropped and Bryonia, 6th, substituted for it, with Nux continued. There was still no effect, and other remedies were tried in alternation with Nux, but all to no purpose, unless it were to make me worse. Certain it was that I became worse under several months of this treatment.

This took me along to the time of going to attend my first course of lectures in Cleveland, in the fall of 1851. There I consulted two or three of the professors, and they all told me to take Nux vomica. Upon telling them I had been taking it several months with no effect excepting to get worse, they said it must be taken stronger and gave me the third. This soon made me much worse than I had ever been. I would go four or five days then without a movement of the bowels, and suffer greatly when they did move. Besides that, other symptoms arose that I had never experienced before, and which were so severe as to alarm me not a little, and show me I must not continue that potency.

From that time on to the succeeding fall, when I went to Philadelphia to attend lectures in the Homœopathic College there, I got along as best I could, sometimes taking remedies and sometimes not, but with no improvement, whether taking medicine or not. Indeed, I grew gradually worse all that year, and reached a condition where I did not have an evacuation from the bowels short of a week or over, and then with great suffering. Arrived at Philadelphia, I consulted two or three of the professors, who all told me to take Nux vomica. Upon telling them I had taken it and how long, they told me to take it stronger. Again I took it in the third potency, but it required only a few days to greatly aggravate my case in every way.

I would then go ten days without any action whatever of the bowels or inclination thereto; and when I did get an evacuation, the stool would be no larger than one's thumb, excessively hard and almost black, and followed with severe suffering for several hours or most of the day. All of this time, too, I had a "sweating of blood" from the axilla; that is, my linen always showed a marked reddish tinge there after a day or two of wear. This is a characteristic symptom of Nux vomica, and showed to what an extent this drug had injured and was still injuring me. I, of course, took no more of it, and gave up all idea of further consulting professors of colleges, who had themselves abandoned all the better teachings of Hahnemann or had never accepted them, and knew so little on these points in comparison with what they ought to have known for their positions.

But for the previous remarkable results I had seen, and have herein recorded in part, and my study of Hahnemann's writings,

I should have abandoned Homœopathy as the "humbug" and "system of quackery" my allopathic preceptor had so vigorously proclaimed it to be. But I knew the truth in therapeutics lay somewhere within its sacred precincts to have worked out such results as I had seen.

With this conviction indelibly stamped upon my mind, I went to reading Hahnemann and Bœnninghausen more attentively than ever before. In the latter's *Therapeutic Pocket Book* I looked up my case with the greatest care for several days, and finally concluded Pulsatilla was the remedy indicated. I then went to Rademacher & Shufe's pharmacy and procured a vial of that remedy in the 200th potency (Jenichen's).

At about 5 P. M., I took one dose of four or five small pellets of it dry upon the tongue, though with the greatest doubts of that potency being able to afford me any relief. The next day, however, to my great surprise, I felt better than before in months. In two or three days my bowels moved much more naturally than they had in a year or over, and with comparatively little suffering. Day after day I improved more and more through the first week. At the end of that time, there not being quite so perceptible an improvement from day to day, and fearing to lose the happy effect that had been started, I took another similar dose of the same remedy. This afforded still more relief, my bowels became quite natural in their movements every other day and without suffering, the red perspiration ceased, and I began to feel like a regenerated man.

Ten days after the second dose, fearing again to lose the effect of the medicine, but with no other reason for it but that fear, and with no *need* for further dosing, I took a third dose of the same remedy and same potency. Then was when I rued it, but had my eyes opened. I never felt worse in all my suffering than the next day after that third dose, and for a week or more after it. Again I went ten days without a movement of the bowels, and then with great suffering, and had all my worst symptoms repeated through the ten days or over before there was much letting up in the aggravation.

Another long search for some other remedy, and for an antidote to Pulsatilla, led me to decide upon Belladonna as that remedy. Of this I finally took one dose, also in the 200th potency, and the relief it afforded was almost as marked, in a day or two, as from the first dose of Pulsatilla. I let the Belladonna act two or three weeks, when a second dose of it was taken and that ended the constipation and all serious symptoms without the need of further medication.

There was no indiscretion in diet, late hours, or cold taken to account for the greatly aggravated symptoms after that third dose of Pulsatilla. My long suffering had made me exceedingly cautious in every way and against everything that could disturb my system, and as great, if not greater, care was then taken, so that the full action of medicine should not be interfered with. Consequently there could be no question that the great aggravation was from that third dose of Pulsatilla.

Moreover, on taking the first dose of it I had no confidence it could do me any good in that potency; had never seen a dose of medicine given in a higher potency than the 30th; so there was no imagination about it to work its first results, as some of our brethren seem so fond of proclaiming in all like cases. Furthermore, at the time of taking the third dose, all the hope and confidence in my nature was aroused and concentrated in the idea that I was going to get a complete and thorough cure of all symptoms from that remedy; so here again, if it were imagination that works cures in these cases, I should have gone on recovering more rapidly than before. All who have suffered can imagine the great disappointment to me in being thrown back to my worst condition when hope was at its highest for entire relief.

Thus, then, did an inexperienced student's confidence in the principles underlying our school, and his faith in the teachings of Hahnemann and Bœnninghausen (which show their truthfulness so clearly, whether we look upon their surface or go their very depths), counteract the wrongs in one case, and show the shallow therapeutic wisdom of a half a dozen or more professors in two medical colleges, who did not hesitate to violate much of Hahnemann's best teachings.

It was the fashion then, as now, to denounce from the lecture platforms the purer teaching of Homœopathy, and also to denounce the few who were then struggling to secure a recognition of the better way. Often did I hear it, even was influenced to some extent by it, and but for my own sufferings from false teachings, and salvation through the higher truths of Hahnemann, might to-day be occupying the lower plane with the throng and denouncing those above. And in conclusion of this point, woe be to the good name of those professors of to-day who fail to teach Hahnemann, at least in the minds of all those of their students who ever do come to a knowledge of the truth.

From that day to this, now over thirty years, I have given as much, or more, thought and observation to the question of repeating doses as to any other one branch of medicine. And from those years of thought, observation, and experience, I have no hesitation in proclaiming it as a *fact* that all the other evils against our school put together have not undermined the confidence of such great numbers of our practitioners in the indications and efficacy of our remedies so much, and retarded the advance of our school and its vastly superior therapeutics so effectually, as has this great error of repeating doses too frequently.

How could it be otherwise? The frequent repetition of doses of the *right* remedy, if that be selected, aggravates the symptoms in the majority of all serious cases of disease, often greatly *aggravates* such cases. Then, when that is done, the physician gets alarmed from seeing his patient growing rapidly worse, and gives another remedy, or perhaps several others. As soon as the patient's system can react and rally from the aggravation of the first medicine, which may be still the only *curative* remedy for the case, he gets better; the doctor attributes the relief to one or more of the subsequent remedies, which have really done nothing in the case, only perhaps to retard it; and his confidence in the first medicine for that combination of symptoms which called correctly for it is gone. Consequently he takes a step back toward empiricism. Numerous failures of a like character confirm him in his first step and hurry him on to others until he is lost in skepticism and empiricism, whereas a dose, or two doses at most, of the *right* remedy would have shown him such marvelous beauties in its curative powers in that same case, and all cases of exactly similar symptoms, that he would cling to his true indications as tenaciously as to his life. Such experience, too, would enable him to help in building up the most important science, that of true homœopathic therapeutics, with which this world has anything to do, and for which it has the greatest need. In contrast with this, how ugly is the reverse side of the picture becoming, and is being made more ugly every day from the would-be artists in our ranks.

From the time of that remarkable relief from the two doses of Pulsatilla, and the equally remarkable aggravation from the third dose of it, to the present day, I have read most of the journals published in our school, and have carefully scanned multitudes of clinical reports of cases treated, and the method pursued by great numbers of physicians reporting on their treatment and its results.

In that time I must have read hundreds of cases where the evidences were clear that the right remedy was given at first, and afforded great relief for a day or two, or a few days in acute cases, or a week to a few weeks in chronic cases; but the doses being repeated every hour or two, or few hours, serious and often alarming symptoms would be reported and the remedy changed. On the contrary, had but one or two doses been given during several days in acute, or many weeks in chronic cases, the physician would have had a brilliant cure to have reported, instead of the dragging and unsatisfactory record that was finally given of the case.

The evidence of aggravations from the right remedy have been so great in large numbers of these cases that I have often wondered the prescriber did not see them himself. And what is a still worse feature of this business is that not a few of these reports have been by our very best and closest prescribers. Indeed, the better the selection of the remedy the more certain the injury from its frequent repetition.

Nor in this general criticism do I spare myself. It was years, ten or twelve of them, before I could bring my confidence up to the point of trusting to a single or but second dose of any remedy in violent acute cases; notwithstanding my personal experiences, and all that I had observed in aggravations in many chronic cases that I had failed to allow my own sufferings to prevent me from overdosing. But when I did get to trusting to the single dose I then began to cure hydrocephalus, malignant diphtheria, and many other malignant forms of disease that I had always before utterly failed to save, and many cases which physicians generally say cannot be cured.

It is from the *palliative* remedies only, as a rule, that beneficial effects can be obtained by repeated doses; and then nature works out the cure herself if the disease is of the self-limiting kind. Whereas, the frequent repetition of the pure curative remedy will certainly bring great aggravations and certain death in many cases of malignant disease that might be saved by great caution and patience in waiting for the single or second dose to develop its action.

Hydrocephalus, malignant diphtheria, malignant typhoid, violent cases of pneumonia, etc., bring the physician face to face with trains of symptoms that palliative remedies can never cure, when he must give the *curative* remedy, and but a dose or two in several days, or see his patient succumb. And here let me say that the true curative remedy in but a dose or two is infinitely the greatest palliative remedy also; that is, it will afford relief from great suffering far quicker, to a much greater degree, and more permanently than is possible with any merely palliative medicine, as is attested by all the severe cases I have reported herein.

And this brings up other points of great importance. In acute sufferings, where the pain is *intense*, if one dose does not relieve in half an hour to an hour or two, it is almost certain that remedy is not indicated and another should be selected. The more intense the suffering, the quicker the relief, generally, from a dose of the right remedy, as I have seen in hundreds of cases. Hence time need not be lost in such cases by waiting several hours for a dose to develop its action. In violent neuralgias, or acute cases generally, with *violent* pains and *little or no inflammation*, we may not be required to wait more than half an hour to an hour, perhaps less, before giving a dose of another remedy if a dose of the one first selected does not relieve; and so of a third and fourth remedy, if the second does not help. But when we come to hydrocephalus, diphtheria, pneumonia, or other established inflammation, then we must wait for the one dose to do its work, and often from one to two or three days, though careful observation ought to, and generally does, show some relief in some one or more prominent symptoms within one to six, twelve, or twenty-four hours. In pleurisy, peritonitis, endocarditis, etc., however, with violent pain, I would not wait but a few hours at most, if there was no relief, before giving a dose of another remedy. If these diseases are not attended by violent pain then we must wait.

It will have been seen that the cases reported in the first part of this paper, and which were attended with great or intense sufferings, even to my father's terrible attack of dysentery, were all relieved of nearly all suffering in ten or fifteen to twenty minutes, and complete cures followed in all of them with little or no more medicine. To these cases large numbers of others could be added showing just as astonishing results from the first dose in many of them, or a second dose in others, or possibly a dose or two of a second remedy. Then why should we not have far more extended trials and observations under this method of prescribing, and thus establish a reliance upon true indications and a confidence in our remedies that time cannot give in the hap-hazard manner these things are carried on now? One dose of vaccine, that is, one insertion of its virus, lasts several years in all, and a lifetime with many; then why cannot we trust one dose of the right medicine for a few hours, days, or weeks, as the case may be, to work out its great results, especially in view of the marvelous cures that have thereby been wrought in thousands of cases, under the observations of different physicians?

In conclusion, I will summarize the rules for dosing as follows:

First. *Never give more than a second dose* of the curative remedy, even in the worst cases of acute diseases, without waiting several days, and perhaps giving a dose or two of some other remedy if the symptoms call for it before returning to the first; and never give more than a second dose of a remedy in the worst cases of chronic diseases without waiting many weeks before giving more of it, and giving a dose or two of another remedy or other remedies if called for.

Second. If a dose or two of a remedy does not give marked relief in from a few minutes to a very few hours under intense acute suffering, or in a day or two in established inflammations, or other acute affections where there is not such intense sufferings, it is not the right medicine. And in chronic diseases, if a remedy does not give perceptible relief in some one or more of its prominent symptoms in from one to twelve or twenty-four hours, if the sufferings are great, or in a few days if not so great, then that remedy may be discarded as almost certainly not indicated. But great care must be exercised, if much pains have been taken in first selecting the remedy, to scrutinize the results, and *know* that it is not acting before it is discarded.

Third. A frequent repetition of the curative remedy will greatly hazard the chances of cure in the majority of serious cases of disease, and certainly so aggravate the symptoms in many cases as to render them either fatal or, in long-standing chronic diseases, incurable under any subsequent treatment.

Fourth. By following these rules and observing very carefully the effects of the first and second doses (if it is thought best to give more than one dose), and discarding that remedy if it does not relieve in the proper time, according to the nature of the case, much valuable time will be saved and the right remedy be found much quicker than by adhering to a drug that does not relieve in the proper time, simply because we think it must be indicated and ought to cure.

Fifth. It must be borne in mind, of course, that in acute diseases, like various fevers, where the disease naturally has a regular course to run, and where there is not severe acute suffering and no immediate danger, if the remedy holds the symptoms in check or prevents violent ones from arising, it may be the exact curative remedy for the case, even though it does not show curative action or marked relief the first few days. If much more severe symptoms do arise, however, or severe pains or other serious sufferings, then the physician may know his remedy is not right and that he must select another. Nature will be his sure guide in these matters, if he will observe closely and allow her to guide him.

Many of the greatest and cleanest cures I have ever made, in the worst forms of both acute and chronic diseases (and they may be stated at hundreds in each), have been wrought by a single dose of one remedy, while many other hundreds of remarkable cures have been secured by one or two doses of two or three remedies that were called for by complications that are liable to arise in the progress of intricate diseases. Not a line, therefore, of the foregoing has been written under the pressure of fancy, prejudice, imagination, or ignorance, but *solely* from *experience* obtained through *thirty years* of the *most careful observation.*

